# Designing and evaluation of E-health educational intervention on students’ physical activity: an application of Pender’s health promotion model

**DOI:** 10.1186/s12889-021-10641-y

**Published:** 2021-04-06

**Authors:** Sahar Sabooteh, Awat Feizi, Parivash Shekarchizadeh, Hossein Shahnazi, Firoozeh Mostafavi

**Affiliations:** 1 Student Research Committee, School of Health, Isfahan University of Medical Sciences, Isfahan, Iran; 2 Department of Biostatistics and Epidemiology, School of Health, Isfahan University of Medical Sciences, Isfahan, Iran; 3 Department of General courses, School of Management and Medical Information Science, Isfahan University of Medical Sciences, Isfahan, Iran; 4 Department of Health Education and Promotion, School of Health, Isfahan University of Medical Sciences, Isfahan, Iran

**Keywords:** Physical activity, Pender model, Software, Web

## Abstract

**Background:**

The present study was conducted to design and evaluate the software and web-based curriculum based on Pender Model in order to promote students’ physical activity.

**Methods:**

This is a quasi-experimental study conducted on 225 eligible students who were randomly divided into two groups of web and software-based intervention and control. The sample size of the study was selected using stratified sampling method. The evaluation was done with pre-test and post-test and follow-up, which were performed immediately two and six months following the intervention. The data were analyzed employing statistical software SPSS using descriptive statistics, chi-square, one way ANOVA, and repeated measures ANOVA.

**Results:**

The obtained results revealed that the level of physical activity after the intervention in the web and software groups significantly increased compared to the control group (*p* < 0.001). Moreover, the mean score of Pender model constructs, immediately two and six months after the intervention, was significantly different in the web and software groups (p < 0.001).

**Conclusion:**

Our results indicated that, providing tailored message based on health promotion model’s constructs has a positive effect on promoting physical activity of students.

**Trial registration:**

Name: Iranian Registry of Clinical Trials. Registration number: IRCT20181009041298N1. Registration date: 2018–12-02 [retrospectively registered].

## Background

Previous systematic reviews and Meta-analyzes have emphasized the role of regular physical activity in the prevention and treatment of a variety of physical and mental diseases [1]. The American College of Medicine reported that inactivity is a major risk factor changeable in chronic diseases [2], and recommends that all healthy adults have moderate physical activity for at least 30 min per day and 5 days per week [3]. Available data show that 31. 1% of the world’s adult population do not follow recommendations for physical activity [1]. Globally, in 2010, about 23% of adults over the age of 18 had inadequate physical activity [4]. The results of a national survey in Iran published by WHO suggested the prevalence of inactivity to be 67.5% [5].

It is of great importance for students to be engaged in health behaviors, including physical activity, as they often face major changes in their lifestyle [3] and these years are associated with the emergence of dangerous health behaviors, such as reduced physical activity [6]. According to the results of experts in Iran, only 10% of students have sufficient physical activity, which is less compared to that in a country like Australia with 52% of participation [7]. Today, most of the behavior change interventions focus on improving physical activity in order to counter the growth rate of chronic diseases. Studies have shown that traditional methods, such as face-to-face interventions, with geographical, time, and cost limitations, do not have the expected effectiveness in this regard [4]. On the other hand, Internet and computer access has increased dramatically in the recent years, with a large proportion of the young population spending most of their time on the computer [8]. For example, the rates of Internet use among adults between 18 and 29 years old in the US and in Iran are respectively 96% [9] and 52.2% [10]. With the increase in the utilization of electronic media for communication and information by students, web and software-based health promotion projects appear to be effective [6]. E-Health is defined as a combination of technologies to facilitate behavior change and health improvement [9], which comprises a vast set of information technology-based education methods, including computer and Internet that make it possible for everyone to learn at any time and place [11]. E-learning has unique benefits, for instance, flexibility and cost-effectiveness [12], which, on top of providing easy access, allows higher quality education, enhances students’ control over the content, time and location of learning, and helps to acquire the necessary knowledge and skills faster than traditional coach-based approaches [13]. The use of videos, photos, and animations along with educational texts leads to a balance in learning and increases interest in learners. In the meantime, the use of educational software with regards to certain features, such as diversity, attractiveness, modernization, and large amount of information storage could provide these conditions for all learners with different levels of talents [14]. The results of Foster in individuals over 16 years old [1], Yu Lua among students [15], and Alley [16] demonstrated positive evidence to support the effects of distance and web interventions for promoting physical activity.

Herein, we chose Pender Health Promotion Model in which personal threat is not considered as the source of motivation. This was due to the above-mentioned factors reported in several studies concerning regular physical activity; they include self-efficacy [17], perceived benefits, and obstacles [18, 19], personal factors [20], prior related behavior [21], and competitive applicants [22]. In addition, given that preventive models have limited benefits to motivate healthy lifestyles, particularly in young adults who often consider themselves not prone to diseases for various reasons, the selected model could be efficient. This model, which is a comprehensive and predictive model for promoting health behaviors, provides a theoretical framework for discovering the factors affecting health promotion behaviors and is a guide for discovering the complex processes that lead individuals to increase health behaviors [22] and show the influence of three groups of factors which directly and indirectly affect the behavior. These factors include personal experiences, behavioral perceptions and emotions (perceived benefits and obstacles, self-efficacy, interpersonal factors, and behavior-related feelings), and behavioral outcomes (commitment to planning and competing priorities and demands) [23].

On the other hand, given that behavior is a process that occurs over time and through a sequence of stages and that individuals are not under the same conditions for physical activity, providing interventions for all individuals without dividing the audience would not provide the desired results. Therefore, being aware of the situation in which individuals can be helpful in designing appropriate intervention processes at the desired stage is important. The pattern of the change stages as a policy provides not only a way to conceptualize the behavior change, but also a basis for evaluating individuals’ readiness for change at five stages for the intervention and planning (pre-contemplation, contemplation, preparation, action, and maintenance) [24]. Therefore, it was used as a basis for determining the content and design of the target media in this study.

In general, the above-mentioned factors and the statistics of physical activity among students [25, 26] necessitate employing new educational methods and evaluating their effectiveness on promoting this issue. However, there are few studies on designing and evaluating the effectiveness of new media-based interventions on health education. The present study, considering the factors affecting physical activity in the young [22, 27], designed educational content and media in the form of web and software and investigated the effectiveness of these two types of education. The study results would help educators to choose the best method and the most appropriate educational media in this target group considering the factors influencing physical activity and novel educational methods.

## Methods

### Study type and participants

This was a quasi-experimental study with randomized control group. The study population comprised students of Isfahan University of Medical Sciences, who were at pre-contemplation, contemplation, and preparation stages based on the stages of change model. They were willing to participate in the study and had no adverse physical or psychological conditions that would limit participation in the project. The study exclusion criteria included unwillingness to continue participation in each of the stages of the study, physical and mental problems during the study and also being in action or maintenance stages. In order to determine the sample size in each study group with a significance level of 5%, statistical power of 80%, and a standardized effect size of at least Δ = 0.5 [12, 28], 62 individuals per group were considered and finally, 75 individuals were considered with a drop probability of 20%. The subjects were selected with stratified random sampling based on volume and allocation in the intervention groups utilizing volume 3 randomized block allocation approach with stratified randomization. Each faculty was considered as a class and according to the number of its students, the sample was randomly selected from the list provided by the researcher in a systematic way, so that in each faculty the number of selected students would be equal. They were then divided into the intervention and control groups.

### Measure

A four-part questionnaire was used in order to collect the data. The initial part examined the background information (age, gender, marital status, residence, and so forth). The second part measured physical activity using the short version of the Persian version of the International Physical Activity Questionnaire (IPAQ), whose validity and reliability have been already confirmed in Iran [29]. This questionnaire measures the level of physical activity in the past seven days and according to the final score, it is classified into three groups: weak, moderate, and severe [29]. In the third part of the questionnaire, Marcus change stage questionnaire [30] was employed for determining our inclusion criteria. The scale consisted of a five-option question in which the individuals could only choose one option according to the physical activity conditions and be placed in one of the five stages of pre-contemplation, contemplation, preparation, action, and maintenance. The reliability and validity of this tool have been confirmed in other studies in Iran [30]. The next part of the Physical Activity Factor Assessment Questionnaire consisted of the Pender Model constructs, which was designed and validated by the research team, in accordance with the stages briefly outlined. The formal validity was assessed by 12 experts in the field of health promotion and the necessary corrections were made based on their views. The content validity was assessed using quantitative methods, and content validity ratio and content validity index for items were evaluated using the opinions of 15 experts. Ultimately, among 208 items, 76 were sufficient. The reliability of the tool was assessed with the internal consistency method, which obtained acceptable Cronbach’s alpha coefficient for all the constructs and the tool (0.865). The external reliability was assessed using intra-cluster correlation indices. The questionnaire was completed by 30 students at two stages with a two-week interval. Subsequently, ICC index was assessed for each construct and the tool that was acceptable (0.846).

### Content and media design

According to the factors affecting physical activity in this age group, previous studies and the results of a cross-sectional study conducted by the researcher prior to this intervention, we primarily provided the content with valid scientific sources within the framework of Pender Model. The program was named “Health to infinity” and based on the fitted model, effective predictors of physical activity behavior and stage, meeting lesson plans, general and specific goals, strategies, and training activities were identified for each session. For the practical part, the desired exercises were first selected, and during the sessions, physical activity and exercise physiology experts assessed the exercises and determined whether they were appropriate for the target group. Following the final modification and confirmation, the filming began and more than 120 videos were prepared in this field. The level of exercise was adjusted; it gradually increased throughout the program, from ten minutes to half an hour at the end of the program. The written text, related images, appropriate music, and videos were used in this training program. The software was designed with C #, sql server database, Adobe Captivate, and Photoshop and the data were stored in a database and prepared as an installer and user program. The program was designed for four to five weeks [14] with a self-regulation booklet. At the beginning of the program, based on Marcus questionnaire, the stage in which the individual is placed (pre-contemplation, contemplation, and preparation) was determined, and accordingly, each person received the appropriate training program. This program was produced as a DVD, and the content used to design the blog. For web, Photoshop was utilized to design the pages, links, and menus. The templates were created with asp.net web design software and C #, and coding software was used to generate the required code. In terms of understanding, matching, and appropriateness to the target group and the attractiveness and applicability of the content, the prepared program was examined by five health education experts and physiologists and two computer and programming experts and their corrective comments were applied. The program was then shown to 15 people in the target groups (students) and their comments were also applied.

### Educational intervention

In this study, there were two intervention groups (1 software group and 1 web group) and 1 control group that did not receive any intervention. In both intervention groups, in the first in-person session, the training was provided regarding the program. In the web group, all the students were asked to visit the blog at least once a day in the next month. In addition, SMS and email were used to inform and introduce the blog. During the intervention, the blog was continuously reviewed and weekly educational content was added to it. In the software group, according to the planned program, the educational content was provided to the subjects for 1 month. In both groups, following the presentation of the program, several activities were planned for the students, which were recorded in a printed booklet. During the course, the participants were able to ask questions from the research team about the program. It was possible to use email to answer questions and problems that the students might have. The evaluation was done with pre-test and post-test, and the follow-up was performed immediately two and six months after the intervention.

### Statistical analysis

After the subjects completed the questionnaires, the data were analyzed with software SPSS 16 (SPSS Inc., Chicago, Illinois, United States). The numerical variables were reported as mean and standard deviation, and non-numerical variables were reported as frequency and percentage. Independent t-test with *P*-value adjustment based on the number of sections was used for comparing the scores of Pender model constructs between the three groups at each time point. Chi-square test and one-way ANOVA were employed to compare respectively the qualitative variables and the three groups. We used repeated measure analysis of variance to evaluate the mean changes in Pender model constructs, and physical activity and covariance analysis to control confounding factors. The normality of the data was assessed with Kolmogorov-Smirnov test and sphericity assumption was evaluated with Mauchly test. The multivariate method was utilized for the evaluation of the effects of time, intervention, and time and intervention interactions once sphericity assumption was not established.

## Results

In the current study, the participants included 115 male students (55.8%) and 91 female students (44.2%). Their mean ages were 23.96 ± 4.94 years and the minimum and maximum age were 18 and 38 years, respectively. Table 1 represents the demographic information of the subjects. Prior to the intervention, no significant differences were observed between the intervention and control groups in terms of the underlying factors, such as age, gender, education, and family economic status (*p* > 0.05).

**Table 1:** Frequency distribution of demographic characteristics in the groups

Demographic Variables	Software		Web		Control		Total		*P.value
	N	%	N	%	N	%	N	%	
**Gender**									0.451
Male	37	53.6	41	62.1	37	52.1	115	55.8	
Female	32	46. 4	25	37.9	34	47.9	91	44.2	
**Marital Status**									0.802
Married	26	38.2	26	40	31	43.7	83	40.7	
Single	42	61.8	39	60	40	56.3	121	59.3	
**Residence**									0.546
Student’s dormitory	38	55. 1	29	43.9	30	42.9	97	47.3	
Student house	9	13	8	12. 1	9	12.9	26	12.7	
With family	22	31.9	29	43.9	31	44.3	82	40	
**Education**									0.930
Bachelor’s degree	34	49.3	30	45.5	35	49.3	99	48. 1	
Master student	14	20.3	14	21.2	17	23.9	45	21.8	
Ph.D student	21	30. 4	22	33.3	19	26.8	62	30. 1	
**Economic Situation**									0.517
Very Well Good	1	1.6	4	6.2	1	1. 4	6	3	
	18	28.6	19	29.2	15	21. 1	52	26. 1	
Medium	40	63.5	38	58.5	51	71.8	129	64.8	
Weak	4	6.3	4	6.2	4	5.6	12	6	

* Chi Squre

* *p* < 0.05

One-way ANOVA test showed that no significant differences were found between the intervention and control groups (*p* = 0.163) before the intervention. Yet immediately two and six months after the intervention, the mean score of physical activity in the intervention groups was significantly different (P _intervention_ < 0.001). Repeated measures ANOVA illustrated a significant interactive effect between the type of educational intervention and time and in the intervention and control groups, the mean score of physical activity was significantly different before the intervention and immediately two and six months after the intervention (${\mathrm{P}}_{{\mathrm{Time}}_{\mathrm{x}}\ \mathrm{intervention}}<0.001$). The trend and time course of changes in all the sections in the three groups were not the same (p _time_ < 0.05) (Table 2).

**Table 2:** The mean score of physical activity of students in the groups

Group	Software	Web	Control	P.value*
	M ± SD	M ± SD	M ± SD	
**Physical activity**				
Before education	420.43 ± 125.04	417.12 ± 126.29	382.352 ± 113.44	P = 0.163
Immediately after the intervention	1693.31 ± 1060.42	1778.78 ± 1762.28	408.97 ± 112.19	*P* < 0.001
2 Months after intervention	3273.91 ± 1125.11	2836.81 ± 1648.07	554.85 ± 655.31	P < 0.001
6 Months after intervention	3011.44 ± 1187.82	2469.69 ± 1284.17	659.26 ± 707.12	P < 0.001
P.value**	P < 0.001	P < 0.001	*P* = 0.015	

Notes: Data are presented as mean & SD

*One-way ANOVA

* repeated measures*

p < 0.05

One-way ANOVA test revealed no significant differences concerning the mean score of knowledge before the intervention between the intervention and control groups, as well as immediately two and six months after the intervention. The comparison of the mean changes in the knowledge scores during the study period between the three groups considering the difference in the level of knowledge at the beginning of the intervention between the studied groups (the knowledge was higher in the control group at the beginning) showed that the effect of this difference as a confounding factor with the analysis of covariance was adjusted with repeated measures. Accordingly, analysis of variance with repeated observations exhibited that the mean knowledge score changes were significantly different between the studied groups (P _intervention_ < 0.001). Furthermore, the mean changes in knowledge scores before the intervention and immediately two and six months after the intervention in all the three groups showed a significant increase (p _time_ < 0.001). The trend of changes in the mean score of knowledge during the study period was significantly different between the three groups (${\mathrm{P}}_{{\mathrm{Time}}_{\mathrm{x}}\ \mathrm{intervention}}$).

One-way ANOVA test implied no significant differences between the mean scores of attitude, which was not significantly different before the intervention between the intervention and control groups, but the mean score of attitude was significantly different immediately two and six months after the intervention between the intervention groups (P _intervention_ < 0.001). The study of the mean changes in the attitude score before and immediately two and six months after the intervention in all the groups showed a significant increase (p _time_ < 0.001). The trend of changes in the mean score of attitudes was significantly different between the three groups (${\mathrm{P}}_{{\mathrm{Time}}_{\mathrm{x}}\mathrm{intervention}}<0.001$) (Table 3).

**Table 3:** The mean score of students’ knowledge and attitude to physical activity in the groups

	Before education	Immediately after the intervention	2 Months after intervention	6 Months after intervention	P_time_^*^	${\mathrm{P}}_{{\mathrm{Time}}_{\mathrm{x}}\mathrm{intervention}}$^*^	P_intervention**_
	M ± SD	M ± SD	M ± SD	M ± SD			
**Knowledge**						P < 0.001	P < 0.001
DVD	16.38 ± 5.21	19.32 ± 5.73	19.32 ± 5.73	19.32 ± 5.73	P < 0.001		
Web	15.64 ± 5.99	18.48 ± 6.87	18.48 ± 6.87	19.12 ± 7.43	P < 0.001		
Control	17. 1± 4.35	18.28 ± 5.23	18.13 ± 5.18	18.97 ± 5.25	P < 0.001		
**Attitude**						P < 0.001	*P* < 0.001
DVD	15.44 ± 6.77	22.7 ± 6.81	22.77 ± 6.79	22.96 ± 6.81	P < 0.001		
Web	15.13 ± 8.36	21.78 ± 8.15	21.85 ± 8.13	21.85 ± 8.15	P < 0.001		
Control	14.53 ± 6.18	15.96 ± 6.05	15.46 ± 6.12	15.17 ± 6.16	P < 0.001		

Notes: Data are presented as mean & SD

* repeated measures

**One-way ANOVA

One-way ANOVA test indicated that the mean score of Pender model constructs, before the intervention, was not significantly different between the intervention and control groups. Yet immediately two and six months following the intervention, the mean score of these constructs was significantly different in the intervention groups (P _intervention_ < 0.001). The mean scores of constructs ahead of the intervention and immediately after two and six months following the intervention in all the groups showed significant changes (p _time_ < 0.001). The trend of changes in the mean score of Pender model constructs was significantly different between the groups during the study (${\mathrm{P}}_{{\mathrm{Time}}_{\mathrm{x}}\mathrm{intervention}}<0.001$) (Table 4).

**Table 4:** The mean score of Pender model constructs before, immediately, 2 and 6 months after intervention in groups

	Groups						
Constructs	Before education	Immediately after the intervention	2 Months after intervention	6 Months after intervention	P*	P*	P**
	M ± SD	M ± SD	M ± SD	M ± SD			
**Prior related behavior**						P < 0.001	P < 0.001
Software	12.37 ± 5.67	5.2 ± 3.13	4.57 ± 2.34	5.16 ± 2.53	P < 0.001		
Web	13.77 ± 6.44	5.2 ± 3.42	4.26 ± 2.02	4.5 ± 2.17	P < 0.001		
Control	12.6± 4.51	12.57 ± 5.27	11.2± 4.87	11.9 ± 5.34	P < 0.001		
**Perceived barriers**						P < 0.001	P < 0.001
Software	30.58 ± 9.81	8.62± 4.38	10.58 ± 5.76	11.46 ± 7.14	P < 0.001		
Web	30.17± 11.42	10.68 ± 8.55	10.24 ± 8. 4	11.38 ± 8.49	P < 0.001		
Control	32.5 ± 7.8	41.93± 22.39	31.81 ± 8.61	30.76 ± 9.28	P < 0.001		
**Situational influences**						P < 0.001	P < 0.001
Software	21.45 ± 6.63	6.46 ± 2.66	7.08 ± 3.31	6.81 ± 3.71	P < 0.001		
Web	21.04 ± 8	6.61 ± 3.9	6.65 ± 3.44	6.17 ± 2.96	P < 0.001		
Control	23.04 ± 5.56	22.72 ± 6.19	22.58 ± 6.14	22.04 ± 6.12	*P* = 0.002		
**Immediate competing demands and preferences**							P < 0.001
Software	28.8 ± 10.19	8.33 ± 3.81	8.65 ± 3.75	10.18± 4.77	P < 0.001	P < 0.001	
Web	29.26± 11. 4	8.16± 4.06	8.69± 4.62	9.57± 4.71	P < 0.001		
Control	32.02 ± 7.77	31.18 ± 8.5	29.64 ± 8.33	28.02 ± 7.97	*P* = 0.002		
**planning**							P < 0.001
Software	7.25 ± 5.46	22.18 ± 6.92	21.21 ± 6.65	21.3 ± 6.68	P < 0.001	P < 0.001	
Web	5.56 ± 3.59	20.73 ± 8.18	19.89 ± 7.75	19.48 ± 7.67	P < 0.001		
Control	5.61± 1.58	6.05 ± 2.01	6.06 ± 2.21	7.85 ± 2.52	P = 0.002		
**Interpersonal influences**						P < 0.001	P < 0.001
Software	9.2± 4.35	23. 4±7.9	21.92 ± 7.39	22.32 ± 7.78	P < 0.001		
Web	7.56 ± 3.77	21.7 ± 8.53	22.16 ± 9.09	22.65 ± 9.15	P < 0.001		
Control	8.33 ± 2.37	8.56 ± 2.68	8.45 ± 2.44	9.29 ± 3.03	P = 0.002		
**Activity–related affect**							
Software	9.58± 4.76	20.93 ± 7. 22	21.64 ± 6.54	16.76 ± 5.37	P < 0.001	P < 0.001	P < 0.001
Web	7.25 ± 3.9	19.69 ± 7.74	19.85 ± 8.24	15.34 ± 6.65	P < 0.001		
Control	10. 1±6. 4	10.32 ± 5.33	10.42 ± 5.95	7.45± 4.62	P < 0.001		
**Commitment**						P < 0.001	P < 0.001
Software	5.57 ± 5.15	18.13 ± 5.47	18.02 ± 5.44	16.89 ± 5.24	P < 0.001		
Web	4.21 ± 3.38	17.29 ± 6.94	17.29 ± 6.5	16 ± 6.51	P < 0.001		
Control	3.78 ± 0.9	3.93± 1.13	3.82± 1.07	5.05± 1.93	P < 0.001		
**Perceived benefits**						P < 0.001	P < 0.001
Software	34.16 ± 10.46	36.58 ± 10.88	35.9 ± 10.82	36.73 ± 10.9	P < 0.001		
Web	30.2 ± 12. 1	34.49 ± 12.93	34.06 ± 12.94	37.42 ± 19.03	P < 0.001		
Control	33.73 ± 8.82	35.6 ± 9.75	33.25 ± 9.66	32.85 ± 9.41	P < 0.001		
**Self-Efficacy**						P < 0.001	P < 0.001
Software	9.54 ± 3.7	34.89 ± 10.61	34.81 ± 10.66	34. 4±10.82	P < 0.001		
Web	8.04 ± 3.35	32.73 ± 12.65	32.84 ± 12.42	32.21 ± 12.31	P < 0.001		
Control	9.66 ± 2.83	10.37 ± 3.53	9.69 ± 3.04	13.45 ± 5.07	P = 0.002		

Notes: Data are presented as mean & SD

*p _Time_

*$$ {\mathrm{P}}_{{\mathrm{Time}}_{\mathrm{x}}\mathrm{intervention}} $$

* Repeated measures

**P _Intervention_

**One-way ANOVA

## Discussion

The present research examined the effectiveness of a web-based intervention and educational software designed to promote students’ physical activity. In this study, physical activity increased significantly following the intervention in the web and software groups compared to the control group. Prior to the intervention in groups of software 2.9% and web 3% had moderate physical activity and none had intense physical activity. After the intervention in the software group, 46. 4% had moderate activity and 47.8% had intense physical activity and in the web group 48.5% had moderate activity and 36. 4% had intense activity. These results confirmed the effectiveness of web and software-based interventions on promoting students’ physical activity, which is consistent with the results of Foster [1], Bell [31], Vandelanotte [32], Hargreaves [33], and Zolfaqari [34]. In Carroll’s study, despite the increase in the physical activity after the intervention, this difference was not statistically significant [35].

Internet behavior change interventions have the potential to reach a large audience at low cost.

The short-term effectiveness of web-based physical activity interventions in previous studies has made participants’ participation and retention a challenge [16]. Fjeldsoe’s study on maintaining a change in physical activity performance suggests that increasing the duration of the intervention or creating long-term follow-up messages in the program might be influential in achieving this goal [36]. In this study, the researcher provided the opportunity to constantly be with the participants and answer their questions by texting and emailing during the six-month intervention and follow-up. In addition, motivating students by showing motivational videos, enhancing self-efficacy, teaching strategies to overcome obstacles, and creating commitment could be regarded as other reasons. Maintaining behaviors by a six-month follow-up for the majority of students confirmed the success of this program. In a study by Maher [37], a significant increase was found in the physical activity after the intervention whereas after three months, it was not significant, which is inconsistent with our study. This issue was tackled with the six-month follow-up of the research team in the present study.

The current work also indicated that those in the software group performed better on physical activity than the web group. The reason could be the easier and faster access to the training program in this group compared to that in the group that needed the Internet to get the program. There are no studies reporting the comparison between these two methods of intervention. Therefore, no comprehensive and reliable comparison is possible between our results and those of previous studies in this regard.

Finding information that supports the behavior changes and knowledge of the undesired outcomes of behavior has always been one of the main processes of change [38] that cannot be ignored. In this study, the mean score of knowledge in all the three intervention and control groups was initially high, which seems reasonable given that our target group included medical students. However, following the intervention in all the groups, the mean score of knowledge increased, which was higher in the intervention groups and statistically significant; this could be attributed to the intervention effect. In the control group, the students’ curiosity to know the answers to their questions and seeking information in this regard could also be the reason of this increase.

Studies have exhibited that improving individual attitudes increases individuals’ participation in the behavior [39]. This study significantly increased students’ attitude score toward physical activity after training in the intervention group by creating a positive interest and attitude in students towards sport activities. The perceived benefits and obstacles of the behavior, specifically at pre-contemplation and contemplation stages, are important for making a change. Therefore, in this program, while focusing on the benefits of regular physical activity, practical solutions were presented to overcome the existing barriers. After the intervention, the perceived benefits in the intervention groups significantly increased and perceived obstacles reduced. This result suggested that the subjects evaluated the benefits and obstacles of the behavior change, considered the change as both feasible and valuable, and were successful [40]. This result is consistent with those of similar studies [41–43].

As a result of the action, the obstacles normally become more pronounced, and less as the behavior continues, and with increasing self-efficacy the behavior is also more likely to be retained. The perceived self-efficacy associated with physical activity was another variable investigated in this study. The exercise self-efficacy, derived from Bandura’s social cognitive theory, expresses one’s beliefs or judgments of one’s ability to perform regular physical activity [44]. Herein, after familiarizing the students with the concept of self-efficacy, various strategies were applied to reinforce it, such as breaking down complex behaviors into small, practical and executable stages, and modeling and rewarding themselves. The results revealed that self-efficacy in the experimental groups increased after the intervention. However, self-efficacy in the control group indicated no significant changes, which is consistent with the results of Lari [43] and Mehdizadeh [45].

The low mean score of action planning before the intervention in all the three groups in this study indicated that the students were not aware of the importance of planning for performing the behavior. In the intervention groups, with emphasis on planning, prioritizing tasks, and writing realistic and achievable goals, the students were taught how to write a good, flexible schedule for regular physical activity. The study results after the intervention showed a significant increase in the mean score of planning in the two intervention groups compared to the control group, which was still high two and six months afterwards.

Studies have shown that the possibility of performing behaviors in which one is admired or socially reinforced has increased [22]. Regarding the low scores of individual factors in all the study groups, several strategies were trained to identify and attract these supports. The obtained findings demonstrated that in the intervention groups, the mean score of individual factors after the training was significantly higher than that in the control group, which is consistent with Lari study [43].

According to our obtained findings, the mean commitment score was low in all the three groups before the intervention. However, immediately after the intervention, the mean score of this construct in the experimental groups was significantly higher than that in the control group. This indicated the effect of the educational program. In this program, emphasizing the role of commitment in the behavior, the students were taught ways to have commitment and were asked to sign and adhere to a commitment made in this regard. A strong commitment to action might save a person from competing demands. The competitive priorities and demands both directly affect the possibility of health behavior and reduce the effect of commitment to action [22]. Given the important role of this construct in the behavior, the students were given training for identifying inactive behaviors and replacing them with regular physical activity. The higher scores of this construct indicated a negative impact on physical activity at the beginning of the study in all the groups. Yet following the intervention, a significant reduction was observed in the intervention groups. According to Bandura, positive or negative feelings are coded before, during, or after the behavior in the mind and once a behavior is initiated in the future it would be reminded. These cognitions and emotions shape the behavior [22]. In this study, higher scores on the behavior-associated emotion constructs implied a good sense of behavior. Ahead of the intervention, it was low in all the groups. The program provided guidance on how to change the way students look and feel about physical activity and help students feel positive about regular physical activity. The results showed a significant increase in the mean score of this construct in the intervention groups after the intervention.

## Conclusion

Since selecting the right educational media in the right model, in addition to increasing the effect on the behavior change, saves time, costs, and resources, and according to the results of the present study, it could be stated that it is time to replace traditional behavior change interventions with modern education methods.

### Limitation

This study was conducted in a single medical university. Thus, generalization of the results to all the medical students in the country is not possible.

## Data Availability

The data that support the findings of this study are available from Deputy of research of Isfahan University of Medical Sciences but restrictions apply to the availability of these data, which were used under license for the current study, and so are not publicly available. Data are however available from the authors upon reasonable request and with permission of Isfahan University of Medical Sciences.

## Abbreviation

IPAQ: Physical Activity Questionnaire
